# Utilization of imaging in the evaluation of pediatric blunt liver and spleen injury: a national trauma data bank analysis

**DOI:** 10.1007/s00383-026-06317-6

**Published:** 2026-02-12

**Authors:** Lia Kwak, Theodore Wang, George Hung, Sathyaprasad Burjonrappa

**Affiliations:** Department of Surgery, Rutgers RWJMS, 1 RWJ Place; Suite 504, MEB, New Brunswick, NJ 08901 USA

**Keywords:** Pediatric blunt liver and spleen injury, Trauma, Operative management, Ultrasound, Computed tomography

## Abstract

**Background:**

The spleen and liver are the most injured organs in pediatric blunt abdominal trauma that can lead to life-threatening hemorrhage. Appropriate imaging via ultrasonography (US) and computed tomography (CT) is essential in identifying the need for operative management in the pediatric blunt liver and spleen injury (BLSI) patients to prevent bleeding complications. Studies have shown increased cancer risks associated with repeated CT use in pediatric patients, but the extent to which CT utilization should be reduced is still unclear. This study aimed to compare pediatric patients who received US only and those who received US followed by CT to determine if imaging modality is associated with clinical outcomes.

**Materials and methods:**

The National Trauma Data Bank (NTDB) was queried for patients ≤ 18 years admitted between 2019–2023 with BLSI who received US and/or CT imaging. Patients with penetrating injuries; concomitant non-abdominal injuries with AIS score ≥ 3; who received CT imaging only; and/or received CT followed by US were excluded. Included patients received US imaging only (US-only), or US followed by CT (US-CT). Baseline characteristics and clinical outcomes were compared between these groups. Primary outcomes measured were incidences of embolization and laparotomy for hemorrhage control. Secondary outcomes measured were mortality; ICU admission; ICU length of stay (LOS); and hospital LOS. Multivariable regression was performed on clinical outcomes with respect to baseline characteristics.

**Results:**

2062 patients met inclusion criteria from 2019 to 2023. Of these patients, 815/2062 (40%) received US only, and 1247/2062 (60%) received US followed by CT. Between groups, no significant differences existed in incidences of embolization (1% vs. 2%, p = 0.20) or laparotomy (3% vs. 3%, p = 0.95). Patients who received US only had a higher incidence of mortality (1% vs. 0%, p < 0.001); shorter ICU LOS (median 2 days vs. 2, p < 0.005); and shorter hospital LOS (3 days vs. 4, p < 0.001). ICU admission was similar between groups (47% vs. 50%, p = 0.24). On multivariable regression analysis, US-CT had no association with ICU admission (OR 0.99, 95% CI 0.70–1.22). Patients presenting to Level I pediatric trauma centers had a lower likelihood of ICU admission (OR 0.58, 95% CI 0.46–0.74).

**Conclusion:**

The addition of CT imaging to US did not appear to affect decision-making for operative management in pediatric BLSI patients. It appears that Level I centers and non-pediatric verified centers have a higher US followed by CT protocol. Further study is needed to determine the use of US and FAST in managing BLSI. Adoption of guidelines emphasizing conservative imaging utilization in pediatric BLSI is necessary to better allocate limited resources.

## Introduction

Abdominal trauma constitutes 10–15% of pediatric trauma cases presenting to hospitals, ranking third most common after head and extremity injuries [1]. Among these, blunt liver and spleen injuries (BLSI) represent the most frequently injured solid organs, with the spleen being the most commonly affected in blunt traumas, followed closely by the liver [2]. These injuries are particularly concerning as they can lead to life-threatening hemorrhage requiring immediate intervention, with current guidelines emphasizing the critical importance of appropriate diagnostic imaging for management decisions.

The management of pediatric BLSI has evolved significantly, with current data showing that over 90% of cases can be successfully managed non-operatively [3]. However, the critical decision between operative and non-operative management relies heavily on accurate diagnostic imaging. While ultrasonography (US) offers a radiation-free initial assessment tool, computed tomography (CT) remains the gold standard for detailed injury characterization.

While CT imaging is frequently utilized in pediatric trauma evaluation, concerns persist regarding the associated increase in cancer risk among children [4]. Furthermore, the rate of CT imaging has been shown to decline with implementation of evidence-based clinical guidelines, with no significant effects to rate of intervention, length of stay, readmission rate, or missed injuries with less CT use [5]. These recent studies raised the question of whether additional CT imaging is essential for surgical decision-making in pediatric abdominal trauma. This study aims to determine whether the addition of CT to initial US imaging significantly impacts decisions regarding operative management. Additionally, we evaluated secondary clinical outcomes to better understand the implications of current imaging practices in the management of pediatric BLSI.

## Methods

The National Trauma Data Bank (NTDB), a voluntary registry supported by the American College of Surgeons (ACS), was queried in this study. The dataset is anonymized and in compliance with the Health Insurance Portability and Accountability Act of 1996 (HIPAA) [7]. Further, NTDB is an incident-based report of voluntarily submitted hospital admission data. Validation rules mitigate submission of missing and non sensical data.

The NTDB was queried for patients aged 18 years or younger with documented BLSI between 2019 and 2023. Liver injury was defined using the following AIS codes: 541,822, 541,812, 541,814, 541,826, 541,840, 541,828, 541,830. Spleen injury was defined using the following AIS codes: 544,212, 544,222, 544,214, 544,226, 544,228. Exclusion criteria included patients who received CT imaging only; no imaging; CT imaging prior to US imaging; penetrating injuries; high-severity non-abdominal injuries; non-isolated BLSI; and missing variables. Patients who received abdominal US and CT were queried from the NTDB using International Classification of Diseases, Tenth Revision Procedural Coding System (ICD-10-PCS) codes BW40, BW41, BW20, BW21, BW24, and BW25. High-severity non-abdominal injuries were defined as having AIS score of 3 or greater and any AIS code that was did not begin with “5.” The remaining patient cohort was divided into patients who received abdominal US (ultrasound) only (US-only group) and patients who received abdominal US followed by a subsequent abdominal CT (computed tomography) scan (US-CT group).

Baseline characteristics including sex, age group, race, ethnicity, vital signs, GCS score, injury severity score (ISS), insurance status, and trauma center verification were analyzed. Age group categories adhered to the Munich Age Classification System, a framework developed for uniform age categories in pediatric emergency care based on pediatric physiology [8]. Vital signs included systolic blood pressure (SBP), pulse rate (PR), respiratory rate (RR), and pulse oximetry. Patients were categorized as having normal or abnormal SBP, PR, and RR based on age-appropriate thresholds [9, 10]. Abnormal pulse oximetry was set conservatively at 95% or lower. Trauma center verification categories involved pediatric level I trauma centers, pediatric level II trauma centers, and non-pediatric verified trauma centers. Primary outcomes included laparotomy for hemorrhage control and embolization. Secondary outcomes included incidence of mortality, ICU admission, ICU length of stay (LOS), and hospital LOS. Readmissions to ICU were included in calculating ICU LOS and were correspondingly reflected in hospital LOS.

Categorical variables were described using counts and percentages. Non-normally distributed continuous variables like ICU LOS and hospital LOS were described using medians and interquartile ranges (IQR). Chi-square analysis was used to determine differences between US-only and US-CT groups in the categorical baseline characteristics, including sex, age category, race, ethnicity, abnormal vitals (i.e., SBP, PR, RR, pulse oximetry), GCS score category, ISS category, organ involvement, insurance status, and trauma center verification. Chi-square analysis was also used for evaluating differences between groups in primary outcomes (i.e., laparotomy, embolization) and secondary outcomes (i.e., mortality rate, ICU admission rate). Fisher’s exact test was used in place of Chi-square test for categorical variables with small sample sizes. Wilcoxon rank sum test was used to determine difference in spread of in ICU LOS and hospital LOS. Multivariable regression was used to determine independent association of baseline characteristics, including imaging modality group (i.e., US-only vs. US-CT) with clinical outcomes described in odds ratios (OR) and 95% confidence intervals (CI). Variable categories with the greatest proportion of patients were established as the reference groups. Statistical significance was defined as p value < 0.05. Statistical analyses were performed in RStudio (version 2024.12.0 + 467).

## Results

### Patient cohort

**Fig. 1:**
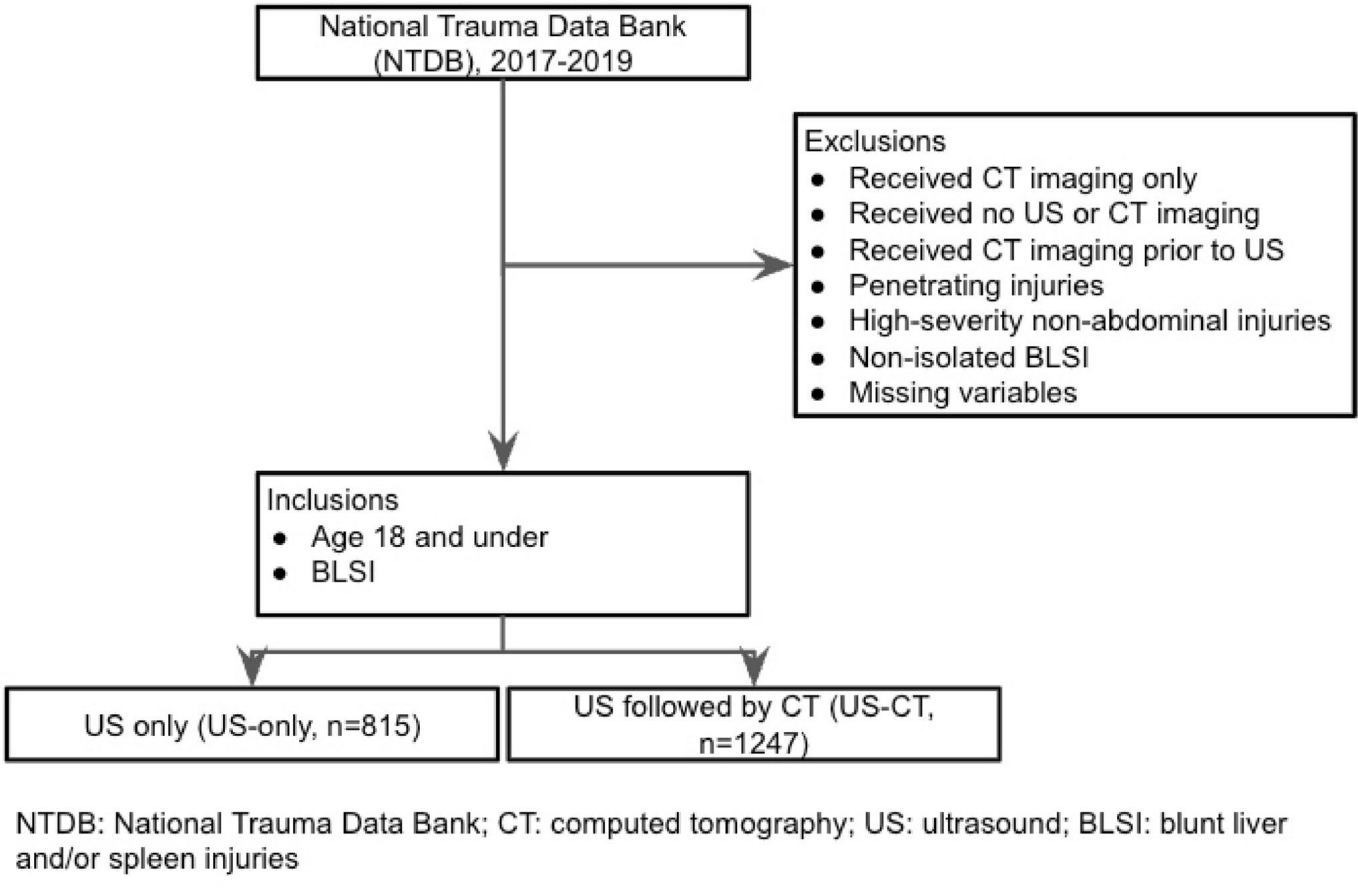
STROBE Guidelines for selection of Cohort for inclusion in this NTDB study

From a total of 8926 patients who presented with isolated BLSI during the study period, 2062 met the inclusion criteria after excluding 6864 patients who received CT only, CT followed by US, or had missing values. Of the included cohort, 815 patients (40%) received US only (US-only), while 1247 (60%) underwent US followed by CT imaging (US-CT) (Fig. 1).

### Baseline characteristics and clinical outcomes

Demographic analysis revealed that males comprised the majority in both groups, with 69% in the US-only group and 63% in the US-CT group (p < 0.01). Patients in the US-CT group trended older in the adolescent age group (12–18 years old [YO], 64% vs. 59%, p < 0.001). Racial distribution showed large variations, with white patients representing the largest proportion in both groups (79% US-only vs. 69% US-CT, p < 0.001).

With respect to vitals, statistically significant differences between US-only and US-CT groups existed only in RR – more patients among the US-CT group had elevated RR (36% vs. 32%, p < 0.01). Hypotension, tachycardia, and low pulse oximetry did not appear to be associated with chosen imaging modality. Mild GCS score (13–15) was associated with US-only patients (98% vs. 96%, p < 0.01). ISS category was not found to be different between groups.

With respect to solid organ involvement patterns, spleen-only injuries were more prevalent in the US-only group (56% vs. 50%, p < 0.01), while combined liver and spleen injuries were more common in the US-CT group (7% vs. 4%, p < 0.01). Liver-only injuries showed no significant difference between the two groups (40% vs. 43%, p < 0.01).

Presentation at non-pediatric verified trauma centers was greater in the US-CT group (60% vs. 42%, p < 0.001). Conversely, more patients in the US-only group presented to Level I pediatric trauma centers compared to those in the US-CT group (40% vs. 25%, p < 0.001). Insurance status was not found to be different between groups (Table 1).

**Table 1:** Comparison of baseline characteristics by imaging modality

	US only (n = 815)	US-CT (n = 1247)	P-value
Sex, count (%)			< 0.01
Male	562 (69)	785 (63)	
Female	253 (31)	462 (37)	
Age, count (%)			< 0.001
Neonate & infant (≤ 1 YO)	2 (< 1)	28 (2)	
Toddler (1–2 YO)	14 (2)	27 (2)	
Early childhood (3–5 YO)	57 (7)	86 (7)	
Late childhood (6–11 YO)	261 (32)	261 (21)	
Adolescent (> 12 YO)	481 (59)	481 (39)	
Race, count (%)			< 0.001
Asian	23 (3)	33 (3)	
Black	59 (7)	185 (15)	
White	645 (79)	862 (69)	
Other	88 (11)	167 (13)	
Ethnicity, count (%)			< 0.001
Hispanic	94 (12)	211 (17)	
Not Hispanic	721 (88)	1036 (83)	
Insurance status, count (%)			0.30
Government	279 (34)	390 (31)	
Private	449 (55)	696 (56)	
Self-pay	44 (5)	87 (7)	
Other	43 (5)	74 (6)	
Trauma center verification, count (%)			< 0.001
Pediatric Level I	327 (40)	315 (25)	
Pediatric Level II	150 (18)	188 (15)	
Adult only	338 (42)	744 (60)	
Systolic BP, count (%)			0.61
Hypotensive	22 (3)	28 (2)	
Not hypotensive	793 (97)	1219 (98)	
Pulse rate, count (%)			0.31
Normal pulse rate	416 (51)	597 (48)	
Tachycardia	384 (47)	630 (50)	
Bradycardia	15 (2)	20 (2)	
Respiratory rate, count (%)			< 0.01
Normal respiration	495 (61)	735 (59)	
Elevated RR	257 (31)	451 (36)	
Depressed RR	63 (8)	61 (5)	
Pulse oximetry, count (%)			0.14
Normal	764 (94)	1146 (92)	
Abnormal (< 96%)	51 (6)	101 (8)	
GCS, count (%)			< 0.01
Mild (13–15)	799 (98)	1198 (96)	
Moderate (9–12)	4 (< 1)	28 (2)	
Severe (3–8)	12 (2)	21 (2)	
ISS, count (%)			0.70
Minor (1–8)	161 (20)	223 (18)	
Moderate (9–15)	366 (45)	562 (45)	
Severe (16–24)	211 (26)	343 (28)	
Very severe (> 24)	77 (9)	119 (10)	
Solid organ involvement, count (%)			< 0.005
Liver only	328 (40)	539 (43)	
Spleen only	456 (56)	627 (50)	
Liver & spleen	31 (4)	81 (7)	

*US* ultrasound, *US-CT* ultrasound followed by computed tomography, *YO* years old, *BP* blood pressure, *RR* respiratory rate, *GCS* Glasgow coma score, *ISS* injury severity score

Treatment outcomes revealed comparable rates of operative intervention between groups. Embolization rates (1% vs. 2%, p = 0.20) and laparotomy rates (3% vs. 3%, p = 0.95) showed no statistically significant differences. However, mortality rate was higher in the US-only group (1% vs. 0%, p < 0.001).

Hospital course analysis demonstrated similar ICU admission rates (47% vs. 50%, p = 0.24). The US-only group was shown to have a greater number of deceased patients (1% vs. 0%, p < 0.05), shorter hospital lengths of stay (median 3 vs. 4 days, p < 0.001), and comparable but statistically significant shorter ICU length of stay (median 2 days for both, p < 0.005) (Table 2).

**Table 2:** Comparison of clinical outcomes by imaging modality

	US only (n = 815)	US-CT (n = 1,247)	P-value
Primary outcomes, count (%)			
Laparotomy for hemorrhage control	23 (3)	37 (3)	0.95
Embolization	11 (1)	28 (2)	0.20
Secondary outcomes			
Discharged deceased, count (%)	8 (1)	0 (0)	< 0.001
ICU admission, count (%)	381 (47)	617 (50)	0.24
ICU LOS, median (IQR)	2 (2, 3)	2 (2, 4)	< 0.005
Hospital LOS, median (IQR)	3 (3, 5)	4 (3, 5)	< 0.001

*US* ultrasound, *US-CT* ultrasound followed by computed tomography, *ICU* intensive care unit, *LOS* length of stay, *IQR* interquartile range

### Multivariable regression analysis of clinical outcomes

Multivariable regression was performed only on ICU admission with respect to baseline characteristics due to small sample sizes in other clinical endpoints (i.e., laparotomy, embolization, mortality). Baseline characteristics associated with a greater likelihood of ICU admission included tachycardia (OR 1.37, 95% CI 1.11–1.70), moderate GCS (score 9–12, OR 2.89, 95% CI 1.19–7.89), moderate ISS (score 9–15, OR 2.33, 95% CI 1.74–3.14), and combined liver and spleen injuries (OR 2.08, 95% CI 1.31–3.37). Patients with severe and very severe ISS scores had expectedly greater odds of ICU admission compared to those with moderate ISS scores.

Imaging modality (i.e., US-only vs. US-CT) had no association with ICU admission (OR 0.99, 95% CI 0.79–1.22). Patients who presented to Level I pediatric verified trauma centers had a lower likelihood of ICU admission (OR 0.58, 95% CI 0.46–0.74) compared to those who presented to non-pediatric verified trauma centers. However, ICU admission in Level II pediatric verified trauma centers trended higher compared to non-pediatric verified trauma centers (OR 1.33, 95% CI 0.99–1.78) (Table 3).

**Table 3:** Multivariable regression analysis of baseline characteristics associated with ICU admission

	OR (95% CI)	P-value
Sex		
Male	Reference group	
Female	0.94 (0.76 – 1.17)	0.60
Age		
Adolescent (> 12 YO)	Reference group	
Neonate & infant (≤ 1 YO)	1.78 (0.75 – 4.52)	0.20
Toddler (1–2 YO)	2.16 (1.06 – 4.57)	0.04
Early childhood (3–5 YO)	1.30 (0.88 – 1.93)	0.19
Late childhood (6–11 YO)	0.77 (0.61 – 0.98)	0.03
Race		
White	Reference group	
Asian	1.76 (0.99 – 3.19)	0.06
Black	1.00 (0.72 – 1.37)	0.98
Other	1.21 (0.87 – 1.68)	0.25
Ethnicity		
Not Hispanic	Reference group	
Hispanic	1.08 (0.79 – 1.47)	0.63
Insurance status		
Private	Reference group	
Government	0.83 (0.66 – 1.05)	0.11
Self-pay	0.69 (0.45 – 1.05)	0.08
Other	0.88 (0.57 – 1.35)	0.56
Trauma center verification		
No pediatric verification	Reference group	
Pediatric Level I	0.58 (0.46 – 0.74)	< 0.001
Pediatric Level II	1.33 (0.99 – 1.78)	0.05
Imaging modality		
US only	Reference group	
US-CT	0.99 (0.79 – 1.22)	0.89
Solid organ involvement		
Spleen only	Reference group	
Liver only	0.84 (0.68 – 1.05)	0.12
Liver & spleen	2.08 (1.31 – 3.37)	< 0.005
Systolic blood pressure		
Not hypotensive	Reference group	
Hypotensive	1.24 (0.65 – 2.40)	0.52
Pulse rate		
Normal PR	Reference group	
Tachycardic	1.37 (1.11 – 1.70)	< 0.005
Bradycardic	1.25 (0.58 – 2.70)	0.56
Respiratory rate		
Normal RR	Reference group	
Elevated RR	0.92 (0.73 – 1.15)	0.46
Depressed RR	0.97 (0.63 – 1.51)	0.90
Pulse oximetry		
Normal SpO2	Reference group	
Decreased SpO2	1.19 (0.82 – 1.73)	0.36
GCS		
Mild (13–15)	Reference group	
Moderate (9–12)	2.89 (1.19 – 7.89)	0.03
Severe (3–8)	1.46 (0.66 – 3.42)	0.36
ISS		
Minor (1–8)	Reference group	
Moderate (9–15)	2.33 (1.74 – 3.14)	< 0.001
Severe (16–24)	5.18 (3.77 – 7.18)	< 0.001
Very severe (≥ 25)	8.14 (5.31 – 12.65)	< 0.001

*OR* odds ratio, *CI* confidence interval, *YO* years old, *PR* pulse rate, *RR* respiratory rate, SpO2 oxygen saturation, *GCS* Glasgow coma score, *ISS* injury severity score, *US* ultrasound, *US-CT* ultrasound followed by computed tomography

## Discussion

Appropriate triage of pediatric BLSI is a sensitive topic. Evidence-based guidelines support non-operative management for the majority of cases, but provider preferences for risk aversion may often outweigh low clinical suspicion when choosing comprehensive imaging, especially in pediatric trauma where risk of uncertainty is not tolerated well.

The goal of this study was to see if there would be a significant difference in operative management and secondary clinical outcomes between patients who received US only and those who US followed by CT. Results showed no significant difference in rates of operative intervention or embolization between the groups. Given the absence of meaningful differences in clinical outcomes, routine CT that follows initial US appears unlikely to change management. Observed variations in baseline characteristics did not translate into differences in clinical endpoints, which further questions the necessity of routine CT imaging following US for pediatric BLSI.

The secondary outcomes also revealed interesting findings. Mortality between the US-only and US-CT groups showed significant difference, with 8 deaths in the US-only group. This is likely explained by selection bias for greater injury severity in patients who received US only, as US is the preferred initial imaging modality in hemodynamically unstable trauma patients. In addition, ICU and hospital length of stay (LOS) were longer in the US-CT group, while the ISS was similar between the two groups. These findings indicate that the addition of CT imaging may lead to increased detection of abnormalities that do not necessarily reflect greater injury severity from the trauma but may prompt a more resource-intensive care with longer monitoring.

Multivariable regression analysis revealed that the addition of CT to US was not associated with increased likelihood of ICU admission. This suggests that additional CT imaging may not influence ICU admission decisions for pediatric BLSI. In a previous NTDB study on pediatric BLSI, Wang et al. previously reported potential overutilization of ICU admission. However, year-over-year analysis revealed a non-significant downward trend of ICU utilization [11]. These findings suggest that, despite best efforts to reduce ICU utilization according to evidence-based guidelines, more opportunity for resource optimization may exist. We argue that reduction of CT imaging in these cases may serve as another opportunity for resource reallocation towards more imminently endangered patients.

Chi-square analysis of baseline characteristics between groups revealed findings that suggest that trauma center pediatric verification status may have a greater influence on management. Of patients in the US-CT group, the majority of patients (60%) presented to non-pediatric verified trauma centers, compared with a smaller majority of patients in the US-only group (42%). Furthermore, on multivariable analysis, presentation to Level I pediatric verified trauma centers was associated with decreased likelihood of ICU admission compared to non-pediatric verified trauma centers. Interestingly, ICU admission likelihood was similar between Level II pediatric trauma centers and non-pediatric verified trauma centers. Given these findings, it is reasonable to believe that appropriate resource utilization can be improved in non-pediatric verified trauma centers where confidence in pediatric triage may be low. Additionally, the pediatric verification process in Level II centers could target imaging utilization or adoption of evidence-based triage algorithms to trend towards rates at Level I centers.

Holmes et al. published a prospective multi-center validation study on the PECARN prediction rules for CT imaging in pediatric emergency department presentations [6]. In this study, 7542 children with blunt abdominal trauma were enrolled and demonstrated high diagnostic accuracy with 100% sensitivity and 100% negative predictive value for choosing the correct tests. The PECARN prediction rules can therefore be a reasonable and safe implementation in trauma centers regardless of pediatric verification status.

Mahdi et al. demonstrated a safe reduction in unnecessary CT imaging in pediatric trauma patients by comparing CT scan rates before and after implementation of an internally developed and validated blunt trauma algorithm at the University of Rochester’s Glisano Children’s Hospital. After implementation, total non-indicated CT scans decreased in all anatomic regions (i.e., head, cervical spine, chest, abdomen, pelvis), cost per patient decreased, and no clinically significant injuries were missed [12]. These findings demonstrate a successful and value-based application of CT imaging stewardship that can be adapted to other institutions.

Additional avenues to explore in future research for determining the diagnostic utility of additional CT imaging include trauma indicators such as reverse shock index x Glasgow coma scale (rSIG), shock index (SI), and shock-index pediatric-adjusted (SIPA). Dante et al. conducted a study that found that prehospital rSIG is reliable predictor of trauma intervention and mortality regardless of injury severity [13]. The rSIG tool may be useful in clinical decision-making to obtain more detailed imaging. Similarly, Yoon et al. conducted a systematic review of the diagnostic utility of SIPA and shock index (SI) in predicting mortality and found that a positive SI can prove useful in identifying high-risk patients and a normal SIPA may identify low-risk patients [14]. Combining different trauma indicators with clinical history and signs may improve existing triage algorithms.

Limitations of this study include those commonly observed in large database retrospective studies such as limited insight into specific reasons for obtaining CT imaging and ICU admission. Additionally, the sample sizes for the other clinical endpoints such as operative management and mortality were too small to confidently identify their predictive variables via multivariable regression. These clinical endpoints are often too multi-factorial to uniformly capture in large database variable sets, so clear-cut explanations of findings are elusive. Furthermore, the NTDB receives data reports from both TQIP (Trauma Quality Improvement Program) and non-TQIP participating Trauma Centers. We obtained the PUF (Participant User File) that provides expanded dataset only from TQIP participating programs. It is possible that there is a selection bias in the data as TQIP participating centers usually tend to be large university hospitals in urban areas. We have mitigated the bias by including data from a diverse group of geographically disparate hospitals in the NTDB and expect that, as with any large database study, we have normalized its content to allow for satisfactory interpretation.

## Conclusion

This retrospective analysis found that additional CT imaging after US did not significantly impact operative management or ICU admission decisions for pediatric BLSI, nor did it identify injuries that increased ISS. Existing pediatric BLSI algorithms may help reduce unnecessary CT imaging, particularly at non-pediatric trauma centers. Further prospective studies are needed to validate optimal imaging protocols.

## Data Availability

No datasets were generated or analysed during the current study.
